# 

**Published:** 1899-02

**Authors:** 


### CONTENTS.

COMMUNICATIONS.

The Innervation of the Tooth-Pulp ........................................ 51
Concerning the Possibilities in the Art of Filling Teeth ................ 59

PROCEEDINGS.

Tri-State Meeting, June, 1898........................................... 71

SELECTIONS.

A New York Oculist ..................................................... 58
The Causation of Chloroform Syncope..................................... 80
Use and Abuse of Hypnotics in Insomnia ................................. 82
The Shrinkage of Amalgams............................................... 84
Milk and Infantile Mortality............................................ 85
Heredity ............................................................... 86
The Effects of Mixed Diets on Salivary Digestion........................ 87
A Bacillus of Scurvy.................................................... 88
White Bread or Brown Bread?............................................. 89
Vaccination and Beauty ................................................. 90
The Origin of Spectacles................................................ 91
Foreign Body in the Throat ............................................. 92
Four Arguments Against Noise............................................ 93
Fahrenheit and Centigrade .............................................. 94
Thymol, as a Succedaneum for Arsenic, in Pulpitis....................... 94
A Mixture to Remove Freckles ........................................... 95
A Registered Dentist Sued .............................................. 96
Long-Delayed Amended Dental Bill ....................................... 96
Tape Worm............................................................... 97

NOTICES.

Fortieth Annual Meeting of the Northern Ohio Dental Association... 97

EDITORIAL.

Care of Children's Teeth ............................................... 98
Regard for Law .........................................................100

OBITUARY.

Dr. F. D. Wilson .......................................................100

